# A new functional classification of U.S. metropolitan and micropolitan areas

**DOI:** 10.1371/journal.pone.0334284

**Published:** 2025-10-17

**Authors:** Robert W. Pendergrass

**Affiliations:** Independent researcher unaffiliated with any institution, Broken Arrow, Oklahoma, United States of America; Science Hub Nepal, NEPAL

## Abstract

This study develops a new functional classification of metropolitan and micropolitan areas in the United States. The methodology used was based on the widely used locational quotient and the Coefficient of Specialization (also known as the Index of Divergence). Determining a specialty or which industrial category may be dominant was set to be the outliers above the upper inner fence for each distribution. The units of analysis were all 927 United States metropolitan and micropolitan areas, excluding Puerto Rico. Readily available employment data from the American Community Survey for 2021 was used. Issues and problems with previous classification systems, such as the reliance on a small number of large cities, the inclusion of unpublished data, and subjectivity, were avoided. A relatively small number of urban areas were found to have multiple functional specializations: only forty-five (4.9% of all areas) had two or more functional specialties. Only five had three functional specialties (just over half a percent). Diversified Metro/Micro Areas, which had no industrial category that stood out as dominant, was the single largest class. The pattern of employment for metropolitan areas that specialized in the Productive Class diverged the most from the overall national pattern of metropolitan areas. Those areas in the Extractive Class followed with the second highest degree of divergence.

## 1. Background

The classification of settlements has a long history in urban geography, urban sociology, and regional economics. There have been many ways of classifying urban areas and human settlements. The most common typology has been based on population. A common typology found is descriptive, ranging from the smallest to the largest: roadside, hamlet, village, town, city, metropolis, and megalopolis. While primarily based on population size, and sometimes on the number of structures, some classifications also bring into the mix the idea of function and purpose. For example, a village with under 1,000 people, at least ten business establishments, and may have an automobile dealership, a feed dispensary, and a grade school [1,6]. Moving away from solely population-based classifications have been functional-based classifications. All such attempts have the same basic foundations but differ in operational definitions and measurement.

This study advances research on this subject by avoiding the problems and issues that have plagued previous urban functional classifications, such as the use of unpublished data, subjectivity, or excluding particular employment groups. This study counters the problems found in previous studies as it is inclusive of all employment categories; it is based on total employment, not labor force; it is inclusive of all metropolitan and micropolitan areas; it does not use unpublished data; all data is readily available and accessible; and the selection criteria are objective. The methodology was based on commonly used locational quotients. Determining a specialty or which industrial category may be dominant was set to be the outliers above the lower outer fence for each distribution.

Several considerations must be disentangled to build and utilize a functional classification. On the theoretical side, these are the meanings of function and purpose and what urban means. On the operational side, the issues are what entity to examine, what indicators to use, what to do about endemic categories, establishing cut-off points, and allowing for multiple specializations. The approaches used in the past assist and highlight these concerns.

### 1.1. Meaning of functional

There are multiple ways the term functional has been used. These diverse ways confuse and blend different concepts when creating a typology. In general, **function refers to what the town does for its residents, immediate surrounding area, region, state, or nation**. Function is tied to why a settlement exists. The first principle commonly used is qualitative in nature and usually refers to cultural importance. A town is functionally important because it is a political, religious, or educational center. However, function does not automatically mean importance or purpose.

The term functional, in urban geography, urban sociology, and regional economics, rests on two interrelated constructs, both of which are tied to economics. First is the **concept of central place**. Walter Christaller first presented central place theory. This theory posits a size hierarchy of places related to the number of establishments (functions) present, which influences the spatial distribution of settlements. The smaller places (lower-order) have fewer functions than larger places (higher-order). There are three interrelated concepts: function, which is any establishment or institution that serves a population; a range, which is the maximum distance people are typically willing to travel to obtain a function; and threshold, which is the minimum size of an agglomeration of people necessary before a function is provided [2]. Each settlement has a tributary area for which it provides services. The larger the settlement, the larger the tributary or service area.

Edward Ullman explained it this way. “Services performed purely for a surrounding area are termed “central” functions by Christaller, and the settlements performing them “central” places. An industry using raw materials imported from outside the local region and shipping its products out of the local area would not constitute a central service.” [3].

Christaller’s definition of centrality (the excess of functions provided by a settlement above what the place’s population needs but is for the tributary region) plugs right into the construct of the **economic base**, the second principle underlying functional classifications.

Thus, any establishment, such as a grocery store, a hardware store, a library, or a manufacturer of automobiles, is a function. The question for central place theory is if it functions only for the settlement, the settlement’s hinterland, or outside its zone of influence. For the economic base, the question is what share of the function serves only the settlement’s population and what share serves the population outside the community. Christaller’s central place activity would be the same as a basic activity.

**Non-basic economic activity** refers to the activity which meets the needs of the place’s inhabitants, the local population. The **basic component of economic activity** of a settlement refers to that portion of the economy that brings in revenue from outside the area either by directly exporting goods or by persons coming into the community from outside its boundaries [4]. It is “basic” to the settlement’s existence. This partially overlaps the central place functions but extends them to areas external to the settlement and its tributary area.

Theoretically, it is necessary to determine the split between basic/non-basic for every establishment. For example, a grocery store will have customers from within and outside the town. This is very impractical. However, the basic/non-basic concept underlies all functional classifications, explicitly or implicitly. As John Alexander noted: “… any functional classification of settlements can be based on the basic functions which do differ from city to city without being confused with functions which are not much different.” [5]

These different uses of the term functional and the blending of the definitions can be found in the typologies created in the past.

### 1.2. Meaning of Urban

Both a theoretical and an operational issue, the question of what urban is has plagued social research due to its consequences on the units of analysis, thus affecting the structure of a study. There are two aspects of “urban” or the “city”: the actual physical footprint of the city and its labor market. The urban area’s physicality is the city’s core underlying perspective, and researchers try to approximate the physical city as best as possible. To be urban, usually, three criteria must be met: population size, density, and physical compactness or contiguousness.

Much research has been on the more significant incorporated places, such as the incorporated City of Chicago, the incorporated City of New York, or the incorporated City of Washington DC. In the past, the legally incorporated area of a city did match the physical city reasonably well. However, continued population growth has far outstripped the political boundaries of most cities.

The best approximation of settlement patterns on the ground is the **Urban Area** as defined by the U.S. Bureau of the Census. It is the best systematic attempt to identify a physical city empirically. Urban Areas ignore all political boundaries. An urban area of 50,000 in population or more (previously termed an Urbanized Area) is the core of all Standard Metropolitan Areas [6]. The Metropolitan Statistical Area (MSA) is a much better approximation of the economic “city” and its labor market, but it can include a large amount of undeveloped land or agricultural land. For example, the Tulsa, Oklahoma metro area has 6,460 square miles, but only 436 square miles are in urbanized areas. The MSA was not designed to account for the physical aspect of a city; it is more of a representation of the labor market and commuting area of the core urban area: its economic footprint.

The metropolitan area becomes a good approximation of the core settlement (the urban area), and its central place hinterland is the balance of the linked surrounding county areas.

### 1.3. Purposes

#### 1.3.1. Purpose of this study.

The purpose of this study was to provide a descriptive analysis and offer a means for simplifying, clarifying, and summarizing complex data related to the enormously complex urban environment, thereby providing a shorthand description of the economic base for cities. The resulting typology then becomes heuristic, providing analytical entities for exploring a broad range of empirical questions. In creating the typology, the following guidelines or principles were prominent: [1] an up-to-date typology; [2] reproducible functional typology of U.S. metropolitan areas; [3] avoid the pitfalls found in previous studies; [5] to make the data and the classification linkable to other socio-economic, health, political or crime data and; [6] to be available for other researchers.

#### 1.3.2. Purposes and issues of typologies.

Any typology can easily have more than one purpose, and all can have issues. and there has been much criticism of city functional classifications. One of which has been that there is no real purpose for one. Robert Smith makes the statement, “… specific geographic objectives---or, for that matter, objectives in general-usually are difficult to discern in the statements of purpose appearing in functional classifications of town.” [7] Richard Warner goes even further, saying, “… these functional classification schemes has seldom furthered our understanding of urban structure or process, since few have been utilized to explain or predict other facets of urban social organization.” [8] Two studies showing the connection to other aspects of urban social structure. Howard Nelson completed one by linking his classification to and finding significant differences in other social attributes such as age structure and educational attainment. [9] Carl Chatters found that differences in functional classification had differences in city taxing strategies and differences in fiscal administration. [10]

However, these concerns can be raised about any typology, classification, or taxonomy, and the criticisms miss the point. It is not necessarily up to the creator of the typology to demonstrate that it is helpful for prediction. Some classification systems have tried to have the classification cover a wide range of subjects, explaining everything. This can have unwanted results. As Tim Brennan comments, “…In attempting to “explain everything,” such systems may also lose descriptive accuracy and become less relevant …” and increases ambiguity. [11]

Thomas Wilkinson provides a purpose: “The assumption underlying [the] usefulness [of an urban functional classification] is a recognition of the interdependence of dominant traits of economic organization in an urban complex and other significant characteristics of its population.” [12] In discussing classification systems in criminology, Tim Brennan provided a list of purposes. The first is a description to clarify complex subjects, simplify and summarize data with minimum loss of information, prevent information overload, discover new or hidden structures, aid in prediction, and test hypotheses and theories. [11]

David Arnold gives several purposes for typologies and classifications: “Classification is no more than an attempt to group items (physical objects, biological characteristics, economic and social data, words, etc.) based on similarities or differences as measured by data.” [13]; “…helps to bring order out of a seemingly incomprehensible mass of national, state, metropolitan, and local data.” [13] and to; “…classification provides the indispensable data framework for continuing analysis.” [13].

The U.S. Census Bureau provides perhaps the most simple and essential purpose: “Classifications serve as a lens through which to view the data they classify.” [14]

Brian Berry sums it succinctly: “If every object in the world were taken as distinct and unique, our perception of the world would disintegrate into complete meaningless. The purpose of classification is to give order to the things we experience. We classify things so that we may learn more about them.” [15]

### 1.4. Approaches used in the past

Aurosseau made one of the earliest systematic efforts at an economic functional classification in 1921. He points out that there is usually one activity that overshadows the rest. He proposed a six-category typology: administrative, defense, culture, production, communication, and recreation [16].

As explained by Ergon Bergel, “… McKenzie bases his classification on economic stages: primary production (agriculture, husbandry, fishing, mining, and lumbering); the second, distribution of primary products; and the third, the manufacture of these products. The fourth category is residual, comprising all noneconomic activities.” [17] Bergel further elaborates these four primary types into five groupings: “… (a) primary “extraction,” mining, fishing, oil production; (b) conversion of raw materials into other goods, manufacture or industry; (c) spatial distribution of goods, transportation; (d) social distribution of goods, wholesale and retailing; or (e) supplementary services, financing, brokerage, advertising, and the independent professional services of lawyers, architects, accountants, and inventors.” [17]

Homer Hoyt presented a classification in 1941. He focused on what needs to be examined in an economic analysis by organizations locating facilities. It was a basic/non-basic employment analysis. The typology used was manufacturing, trade and finance, extraction, resorts, political capitals, higher education, and transportation. All other employment categories, such as retail and most services, were considered non-basic employment. [18]

Chauncy Harris developed a typology in 1943 based more on empirical data than previous systems, though he combined empirical data with the subjective [19]. His classification was based primarily on employment data from the U.S. Census but was supplemented with data on occupation. Other data, such as university enrollment, were also considered. When determining which category a city would be in, Harris only considered the employment as a percentage of the employment in Manufacturing, wholesaling, and retailing, not the city’s total employment. The percentages varied from industry to industry.

In a study of cities in the American South, John Hart replicated Harris’s classification, though he used only occupational data [20] to explore how cities changed over time. Ten occupational groups were used, and percentages were based on the total labor force. The cut-off points were set to the upper decile for each category. These values were set slightly higher for wholesale, educational, and mining centers and slightly lower for professional centers. For military centers, the cut-off was set to ten percent of the entire population. Cities not meeting these cut-offs were deemed diversified. Of interest, Hart considered the minimum percentages for each employment category as the minimum required by cities to be viable [20]. This minimum would be classed as non-basic employment.

Victor Jones, in 1953, presented a multifaceted classification of cities with over 10,000 in population [21]. While metro areas were mentioned, no data or classification was presented for them. He first determined the city’s status as the central city, a suburb, or independent. These were then subclassified as being employing, balanced, or dormitory. He also presented a classification based on rents and another based on the age of the housing stock. The classification of the economic base used multiple data sources: the Census of Manufactures, the Census of Business, and the Decennial Census. The study used labor force. The focus was on manufacturing and trade industries, with several employment categories excluded, particularly all the service industries, though services are included elsewhere. The selection criteria varied for each category without explaining the basis for the cut-off percentages used. The base for calculating the percentages changed from one category to another.

The classification developed by Howard Nelson in 1955 is the most well-known and the most widely used. Ten categories were left out: agriculture, forestry, fisheries, construction, utilities, business services, and private households. Military employment was included in public administration. Proportions were based on the total labor force and not the total employed. The criteria were applied uniformly across the employment categories. In choosing which industries were significant, Nelson used standard deviations. An industry had to be at least one standard deviation from the overall industrial mean. The classification of cities indicated which standard deviation applied: Mf, Mf2, or Mf3. Cities with no activity category one standard deviation or higher were classified as diversified. [22] The system allowed for multiple specializations.

Bergel, in 1955, presented a broad classification, utilized a combination of empirical and subjective criteria, and attempted to make the typology more universal and international [17]. Bergel combined economic, cultural, and political factors. The top-level categories were economic centers, political centers, cultural centers, recreational centers, residential cities, retirement cities, symbolic cities, and diversified cities.

In an exhaustive analysis by Otis Duncan and others, the attempt was to blend and merge economic base concepts, functional classification, and central place theory. The system used was extraordinarily complex and very data intensive. They relied heavily on input-out analysis tables. Underlying the system were two principles: a relationship to resource extraction and a market for outputs. They produced twelve categories within which Census employment categories were fitted. [23]

Categories two through seven were all manufacturing so a larger categorization would be Extraction, Manufacturing, and Services. The categories they used make a comparison to any other typology impossible. Additionally, they introduce a population size hierarchy into the analysis. Analysis was based on location quotients.

A second classification was introduced, which had little explanation on how the classification was constructed other than that value-added, wholesale receipts, and other Federal Reserve data were used. Metropolitan functions appear to be trade functions. [23]

Thomas Wilkerson created an interesting classification of Japanese cities in 1964 [12]. Several issues of international functional classification become apparent, such as differences in available data and definitions of what urban is. Wilkerson examined the male labor force in incorporated cities in 1920, 1930, 1950, and 1955. He developed six categories. The criteria for determining which Class a city was in were different from those used by other researchers: Both mining and agriculture had to have at least 25% of the male labor force employed; for industrial, the criteria was the percent employed in an industry divided by the sum of the percents of commerce, transportation, and administrative employment; for commerce, the criteria was the percent employed in commerce divided by the sum of the percents in industrial, transportation and administrative employment; for transportation and communication the criteria was the percent employed in transportation divided by the sum of the percents in industrial, commerce, and administrative employment; and for administrative and services, the criteria was the percent employed in transportation divided by the sum of the percent of industrial, commerce, and transportation employment. If an individual city had a ratio for a category one or more times found for the total urban employment, it was assigned as a specialty. [12] His system allowed for multiple specializations.

**Fig 1:**
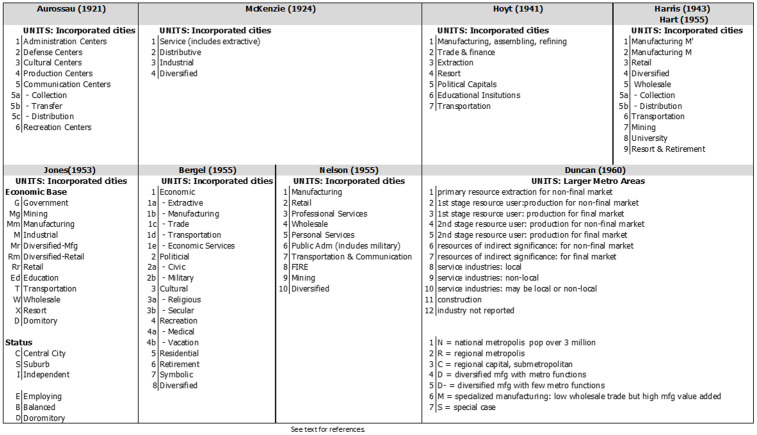
Historical Functional Classifications.

Fig 1 summarizes the classifications from the above studies.

The discussion of past classifications highlights several problems/issues with functional classifications.

- Some employment categories were excluded.
- Labor force was used, not total employment.
- Employment data was based on occupational data.
- The units of analysis were limited to just large cities or large metropolitan areas.
- Data that was/is not readily accessible was used.
- Unpublished data sources were used.
- Subjectivity was endemic to most of the classification systems.
- Differentiating selection criteria varied within studies, often without explanation as to why.

This study counters the problems found in previous studies: it is inclusive of all employment categories; it is based on total employment, not labor force; it is inclusive of all metropolitan and micropolitan areas; it does not use unpublished data; all data is readily available and accessible; and the selection criteria are objective.

## 2. Data and methods

### 2.1. Units of analysis

The units of analysis were all the Metropolitan Areas (MA) and Micropolitan Areas (MI), as defined by the U.S. Bureau of the Census for 2020. A Metropolitan Area has a core country with an Urbanized Area of at least 50,000 people and a Micro Area with an urban area of at least 10,000 people but less than 50,000. Adjacent whole counties can be included if 25% of its workers are employed in the core county. There may or may not be additional Urban Clusters within the counties. Counties not including the primary urbanized areas must be linked to the central county through commuting patterns. The concept is that the Metro and Micro areas represent the economic city. They include physical areas and population which are not urban and thus include rural and agriculture. This causes a conundrum in the employment data discussed in the following section.

In 2020, there were 927 MAs and MIs excluding Puerto Rico: 384 Metropolitan Areas and 543 Micropolitan Areas. The smallest land area was the Metro Area of Vineyard, MA, at 103 square miles, and the largest was the Riverside-San Bernardino-Ontario, CA, Metro Area, at 27,300 square miles. The metropolitan and micropolitan area boundaries were downloaded from tlgdb_2021_a_us_nationgeo.gdb (accessed on 30 April 2022) [24]. The TIGER shapes were converted into points for use in mapping and display.

### 2.2. The data

Data on employment and occupation have evolved. There was a shift away from linking occupation and industry, and until 1940, it was possible to derive the type of business from occupational data as occupations were grouped based on industry. This was no longer true, starting with the 1950 Census [25]. Occupation is based upon the work performed and not the industry in which it is performed. Occupation was standardized into the Standard Occupation Classification (SOC) [25] in the 1970s. Industry was classified by the Standard Industrial Classification (SIC) code list. In the 1990s, this was revised and realigned with NAICS (North American Industrial Classification System), based on the production process of goods and services. This is a unifying feature of the NAICs classification. It is supply-based and production-oriented. [14] The groupings are producing units, not products or services. The bottom-line unit is a physical establishment. NAICs divides the economy into twenty sectors. There are 1,057 industries [26]. This resulted in a list of 236 detailed categories. For 2017, there were twenty major industry sectors. This is further regrouped into fourteen major categories for reporting in the American Community Survey. [25]

The breaking of the link between occupation and industry, plus the changes in classifying industry over time, renders it impossible to make comparisons over time.

The data used was employment by industry from the United States Bureau of the Census American Community Survey for 2021 [27]. Data on occupation was not used. There are 14 mutually exclusive categories [14]:

1. Military
2. Agriculture, forestry, Fishing and Hunting, and Mining (Sectors 11 and 21).
3. Construction. (Sector 23).
4. Manufacturing (Sectors 31–33).
5. Wholesale Trade (Sector 42)
6. Retail trade (Sectors 44–45).
7. Transportation and Warehousing, and Utilities (Sectors 48–49 and Sector 22).
8. Information (Sector 51)
9. Finance and Insurance, and Real Estate and rental and leasing (Sector 52 and 53).
10. Professional, Scientific, Management, Administrative, and Waste Management Services (Sectors 54, 55, and 56).
11. Educational Services, Health Care, and Social Assistance (Sector 61 and 62).
12. Arts, entertainment, Recreation, and Accommodation and Food Services (Sector 71 and 72).
13. Other Services, except Public Administration (Sector 81).
14. Public Administration (Sector 92).

See S2 Appendix NAICS Employment Categories for more descriptive details.

Only 895 of the Metro/micro areas had employment data reported in the 2001 American Community Survey. Only 513 had military employment reported. For this study, zeros were entered for military employment in areas with no reported military employment.

### 2.3. Methods

The methodological issue has been determining when employment in a category is significant, which is dominant, or what the cut-off point should be. Roy Kass reported in 1973 that there were five approaches: (1) based on absolute constraints; (2) based on the normal distribution; (3) based on the minimally necessary level of activity for a community’s viability; (4) based on the relative proportion of activity; and (5) based on a profile of the community [26]. Excluding studies based on subjective criteria, the studies based on objective criteria have used a variation of one of these three methods. Alexander notes that there are only three objective systems.

The first method classifies a city based on the three most extensive activities within a city. For example, in 2021, the New York Metropolitan Area’s most prominent employment category was Educational and Health Services (27.5%), followed by professional services (14.8%) and retail trade (9.6%).

The second set of methods uses positive differentials, usually from a national average. The first and most basic technique is subtracting the national average from the entity’s percentage. The results are different for the New York Metro Area. The top category now becomes professional services (a differential of 6.5%). Second becomes financial services (a differential of 4.4%), and third becomes educational services (a differential of 3.5%).

A second technique within the set of positive differential methods is formalizing the process using standard deviations. This was the technique used in the Nelson classification discussed above. New York City was classified as F2, two standard deviations for finance. The employment data used in this study were converted to z-scores. Using 2021 data, the most distinct category for the New York MA was information (z-score of 2.6). Next was financial services (z-score of 2.1). Professional services were third (z-score 1.9).

The problem with this technique is the use of the statistical mean. Extreme values, low or high, highly influence it. The distributions of the MAs within the employment categories were abnormal, so using statistics that require the normality assumption becomes problematic [7].

The third technique within this set of positive differential methodologies uses location quotients (LQ). These were used extensively in the classifications used by Duncan [8,23,28]. The location quotient calculates the metro area’s proportion of its total employment in each employment category by the same proportion for a larger area, in this case, national metro/micro total employment. For example, the New York metro area had 27.5 percent of its total employment in education and health services. For all Metro/micro areas, the proportion was 23.3 percent. Thus, the location quotient was 1.18. For information services, the LQ was 1.71. Ignoring the issue of cut-off points for now, New York had six categories of employment with Location quotients greater than one: information (1.71), FIRE (1.34), Professional (1.18); Educational and medical (1.18); Transportation and Warehousing (1.06); Wholesale Trade (1.03).

The accepted practice for interpretation is that if the LQ is one or less, the employment is classed as local or non-basic. Over one indicates a more significant share than the national [1,28] and is termed basic employment. Higher breakpoints have been used, including 1.25 and 1.5.

A third set of methodologies uses factor, discriminant, or cluster analysis. Roy Kass completed one such study in 1973 using cluster analysis of location quotients. Kass looked at the entire configuration of location quotients for a metropolitan area. The effort was to find those areas that had commonalities. The cluster analysis procedure produced twelve categories. Our example of New York was classified as retail and finance. In a 1977 study, Richard Wanner also employed factor and discriminant analyses to test two fundamental dimensions of large urban places – an industrial structure and a finance-commercial structure [8].

Tim Brennan also warned, “A danger is that the easy availability of computer algorithms may encourage thoughtless or inappropriate use of complex classification methods to produce a confusing mass of nonconverging and perhaps artificial empirical typologies.” [11]

This study used the location quotient to determine specialization, with the base for comparison being the total employment for all metropolitan and micropolitan areas, not the mean. The equation is:


$$LQ=\frac{\left(\text{Metro Area Employment in Industry i}/\text{Total Metro Area Employment}\right)}{\left(\text{All Metro Area Employment in Industry i}/\text{All Metro Area total employment}\right)}$$

To continue with the New York Metro Area example of information services:


$$LQ=\frac{\left(\text{301},\text{419}/\text{9},\text{373},\text{178}\right)}{\left(\text{2},\text{819},\text{116}/\text{149},\text{924},\text{046}\right)}=\frac{\text{3}.\text{216}\%}{\text{1}.\text{880}\%}=\text{1}.\text{7}\text{1}$$

For this example, the proportion of information services in the New York metro area was 1.7 times the national proportion. In contrast, the highest LQ for information services nationally was 3.5 for the San Jose-Sunnyvale-Santa Clara, CA Metro Area.

**Fig 2:**
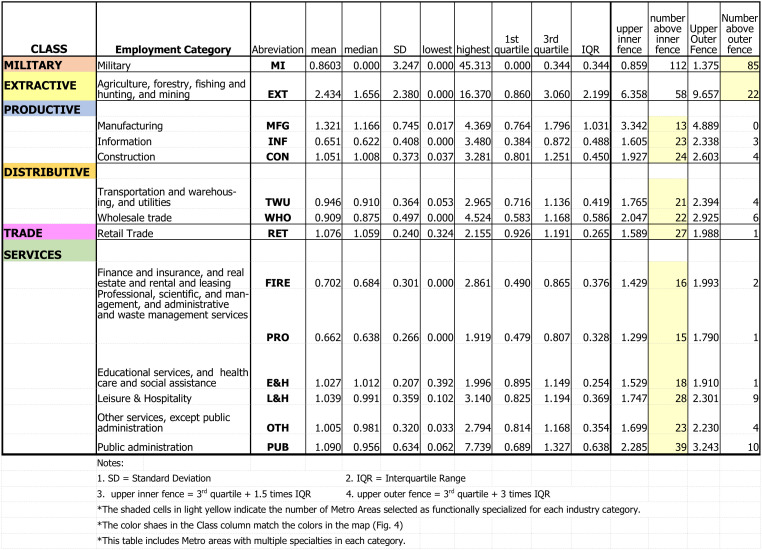
Location Quotient Descriptive Statistics for the Fourteen Employment Categories, 2021.

The selection criteria varied according to the data distribution for the industry category. The differentiating criteria were based on outliers to determine which activity stood out or was dominant. This is like using standard deviations but avoids the pitfalls of relying on the mean. Extractive, Military, and Construction criteria were set to outliers above the upper outer fence. The value for the upper outer fence is set to the third quartile plus three times the Interquartile Range for each of these employment categories. The criteria for the other eleven categories were set to outliers above the lower outer fence, which is the third quartile plus 1.5 times the interquartile range. These selection cut-off points are presented in Fig 2.

For example, the selection cut-off for Extractive industries was a location quotient greater than 9.66. For Military employment, the cut-off criterion was a location quotient greater than 1.38. For Construction, the selection criterion was a location quotient greater than 2.6. For other industrial categories, the selection criterion was greater than the upper-lower fence. Examples include Manufacturing, set to 3.34; Retail Trade, set to 1.59; and Public Administration, set to 2.29. See Fig 2 for a list of all the selection criteria for the fourteen categories.

The Coefficient of Specialization (COS), also known as the Index of Dissimilarity or the Index of Divergence, was determined to get generalizations about a metro/micro area’s overall distribution of industries. Duncan [24] and Isard [30] discussed the COS. The COS “… measures the extent to which the distribution of employment by industry classes in the given region deviates from such distribution for the United States.” [29], though here, the total distribution was for all metro/micro areas. It shows the divergence of the metro area structure from the national structure. The higher the value, the greater the dissimilarity, or divergence, between the metro area and the national.

The coefficient is calculated for each metropolitan/micropolitan area for each industrial category by subtracting the area’s proportion from the total national proportion, summing either the negative or the positive differences for the area.


$$\text{COS}=\sum_{\text{1}}^{C}\frac{\text{1}}{\text{2}}|\frac{E_{ia}}{E_{a}}-\frac{E_{in}}{E_{n}}|$$

E = employment

i = industry category i

a = metro area

n = total national

C = number of categories

The software used was Microsoft Excel (with the add-ins StatistiXL [30] and AbleBits [31]), Microsoft Access, and ArcGIS Pro.

## 3. Findings

### 3.1. Classification schema

A general theme throughout the past research was the flow of material through the economy in stages. The overall categorical structure presented by Ergon Bergel in discussing Roderick McKenzie’s system forms the kernel of top-level categorization, or Class, used in this study after adjusting for the newer employment categorization and building on the NAICs classification and modifying it to fit the task of classifying metro areas. The classification schema used in this study was Military, Extractive, Manufacturing, Distributive, Trade, Services, Multiple, and Diversified. These eight classes are discussed in the following sections (Table 1).

**Table 1:** The Functional Classification Schema.

CLASS	CENTER
Multiple	
Military	
Extractive	
Productive	Manufacturing
	Information
	Construction
Distributive	Wholesaling
	Transportation & Utilities
Trade	Retail Trade
Service	FIRE
	Professional & Business Services
	Educational & Health
	Leisure & Hospitality
	Other
	Public Administration
Diversified	

### 3.2. General

Fig 2 summarizes the descriptive statistics (mean, median, lowest, highest, first and third quartiles, and the interquartile range) for the location quotients for each industrial category. The military had the highest location quotient, recording a value of 45.3 for an equivalent local percentage of 38.1%. The next highest location quotient was 16.4 for the extractive industries. The manufacturing sector registered a median location quotient of 1.2, with the highest being 4.4 (local equivalent of 42.8%). Five industrial categories had zero as their lowest location quotient (military, extractive, information, FIRE, and professional services). The largest interquartile range (IQR) was for the extractive industries at 2.2, followed by manufacturing with an IQR of 1.03. The extractive industries had the largest median location quotient of 1.7, and Manufacturing had a median location quotient of 1.2. The lowest median location quotient (excluding the military which was zero) was 0.6 for the information sector.

**Fig 3:**
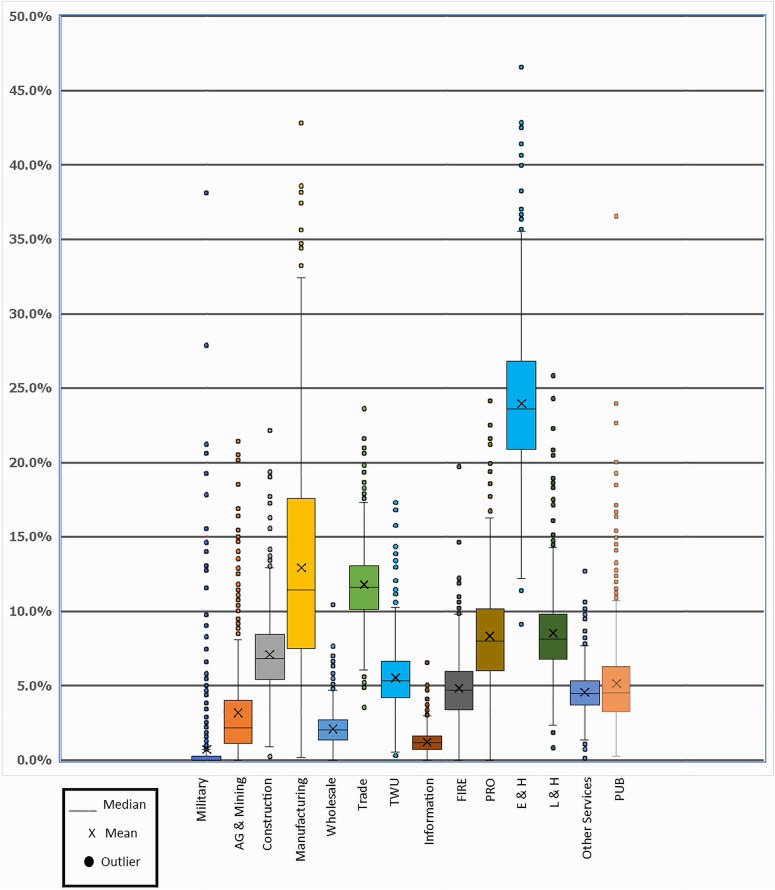
Distribution of Metro and Micro Areas for Each Employment Category, 2021.

The distributions of the metropolitan/micropolitan areas within the categories were far from normal, as can be seen graphically with the box-whisker plots (Fig 3). The distributions were either very skewed, had excess kurtosis, or both. A normal distribution would have a kurtosis value around three and zero skew. For example, the military category had a skew of 7.06 and a kurtosis of 65.2. This means there was a high peak and an extremely long tail. Public administration was another example, with a skew of 2.89 and a kurtosis of 18.15. Nine of the fourteen categories had excessive kurtosis with a value over three. All had a positive skew, and nine were highly skewed.

Some metro/micro areas reported no employment in some functions excluding the military sector. Fifteen metro areas had no employment reported in wholesale trade functions. Forty metro areas reported no employment in information functions. Only one area reported zero in FIRE. Only one reported zero in professional and only one in extraction functions.

### 3.3. Multiple specialization class

The observation that stands out the most dynamically is that not many areas have multiple functional specializations. As can be seen in Table 2, only forty-five areas out of the 927 Metropolitan and Micropolitan areas (4.9%) had two or more specializations. Only five (just over half a percent) had three specializations: Elk City, OK; Espanola, NM; Seattle, WA; Sterling, CO; and Washington, DC.

**Table 2:** Metropolitan & Micropolitan Areas Which Have Multiple Specializations, 2021.

Areas With 3 Functional Specializations	Specializations		
	#1	#2	#3
Elk City, OK	WHO	TWU	L&H
Española, NM	L&H	INF	PRO
Seattle-Tacoma-Bellevue, WA	INF	MI	PRO
Sterling, CO	PUB	L&H	INF
Washington-Arlington-Alexandria, DC-VA-MD-WV	PUB	MI	PRO
Areas With 2 Functional Specializations			
Boulder, CO	INF	PRO	
Breckenridge, CO	L&H	FIRE	
California-Lexington Park, MD	MI	PUB	
Carson City, NV	PUB	L&H	
Cheyenne, WY	MI	PUB	
Cornelia, GA	INF	WHO	
Denver-Aurora-Lakewood, CO	INF	PRO	
Dumas, TX	MFG	L&H	
Fallon, NV	PUB	INF	
Fort Leonard Wood, MO	PUB	L&H	
Fredericksburg, TX	CON	FIRE	
Greenwood, MS	PUB	RET	
Hanford-Corcoran, CA	EXT	MI	
Hobbs, NM	EXT	TWU	
JHacksonville, FL	MI	OTH	
Jamestown, ND	WHO	INF	
Jasper, AL	TWU	RET	
Key West, FL	MI	L&H	
Kirksville, MO	WHO	E&H	
Las Vegas, NM	RET	FIRE	
Mitchell, SD	OTH	TWU	
Newport, TN	WHO	L&H	
Nogales, AZ	PUB	WHO	
Olympia-Lacey-Tumwater, WA	MI	PUB	
Ozark, AL	L&H	TWU	
Palestine, TX	PUB	RET	
Pinehurst-Southern Pines, NC	MI	OTH	
Pullman, WA	INF	L&H	
Rio Grande City-Roma, TX	CON	L&H	
Salinas, CA	EXT	MI	
San Francisco-Oakland-Berkeley, CA	INF	PRO	
San Jose-Sunnyvale-Santa Clara, CA	INF	PRO	
Santa Fe, NM	PUB	PRO	
Sierra Vista-Douglas, AZ	MI	PUB	
St. Marys, GA	TWU	L&H	
Susanville, CA	PUB	INF	
The Villages, FL	CON	OTH	
Vineyard Haven, MA	INF	PRO	
Warner Robins, GA	MI	PUB	
West Plains, MO	WHO	L&H	

Notes: See Fig 2 for abbreviations.

The presence of Washington, DC, on this list is not surprising, as it is the U.S. capital. Public administration had a location quotient of 2.7, almost three times the national percentage in public administration. The military had a location quotient of 1.81, while not exceptionally high, and it was still over the upper outer fence cut-off (1.38) for military employment. Combined, these three categories totaled 36.82% of DC’s employment. Seattle, Washington, also was not a major surprise. Information industries (economic sector 51) include industries that produce and distribute information and cultural products or transmit and process data. Publishing and printing industries are found here, as are broadcasting, telecommunications, and data processing centers such as a GOOGLE Data Center [14], which had a location quotient of 1.8, with the upper inner fence cut-off of 1.6. The military industry also had a concentration with an LQ of 1.7. Professional services (economic sectors 54, 55, and 56, including accounting, mapping, architecture, engineering, consulting, veterinary, and legal services [14]) had an LQ of 1.41, while the upper inner fence was 1.3. A total of 22.54% of the metro areas’ employment was in these categories.

The other three on the list were intriguing. For Espanola, New Mexico, L & H (arts, entertainment, recreation, accommodation, and food services) had a location quotient of 1.79 (the cut-off was 1.7). This is economic sectors 71 and 72. Included are establishments that provide live performances, operate cultural facilities, operate casinos, food services, and operate establishments for travel lodging and recreational lodging [14]. Professional Services had a location quotient of 1.4. Information services had a location quotient of 1.7. These latter two categories are not surprising as this is the home of the Los Alamos National Laboratories. A total of 35.81% of Espanola’s employment was in these three categories.

For Elk City, Oklahoma, wholesale trade had a location quotient of 4.5, the highest LQ for wholesaling in the country. Next in line was TWU (transportation, warehousing, and utilities), with an LQ of 2.7. TWU is industrial sectors 22, 48, and 49, including establishments that provide transportation for passengers or cargo by any means, including pipelines, warehousing or storage, electricity, and water supply [14]. Third was L & H, with a location quotient of 2.13. These categories garnered 43.74% of the metro’s total employment. For Sterling, Colorado, public administration had a location quotient of 3.27. Sterling is the location of a state prison. L & H was next with a location quotient of 2.30. Information industries were third with an LQ of 2.08. A total of 38.29% of the metro’s total employment was in these three categories.

Manufacturing industries only showed up once for the forty-five areas with two or more specialties. This was Dumas, Texas, specializing in meat processing, with a manufacturing location quotient of 3.57. The most common industry was public administration, with fifteen areas having them as one of their two specialties. The second most frequent industry was information, with thirteen areas having it as one of their two specialties. The third most frequent industry was the military, with eleven areas. Tied for third were Leisure and Hospitality specializations. Interestingly, three areas had extractive industries (agriculture and mining) as one of their two specialties.

There appears to be no consistency in the pairings. The most frequent pairing was between information industries and professional service industries, which occurred together in six areas. The second most frequent pairing was the military and public administration, with five areas.

The forty-five areas of the Multiple Class had a median COS of 0.221; 22.1% of those employed would have to change employment categories to match the national distribution. The Class had a range of 0.3064 between the low value of 0.088 and the maximum of 0.394. The metro area with the highest COS was Dumas, Texas. The multi-Class metro area with the lowest value was Seattle, Washington.

**Fig 4:**
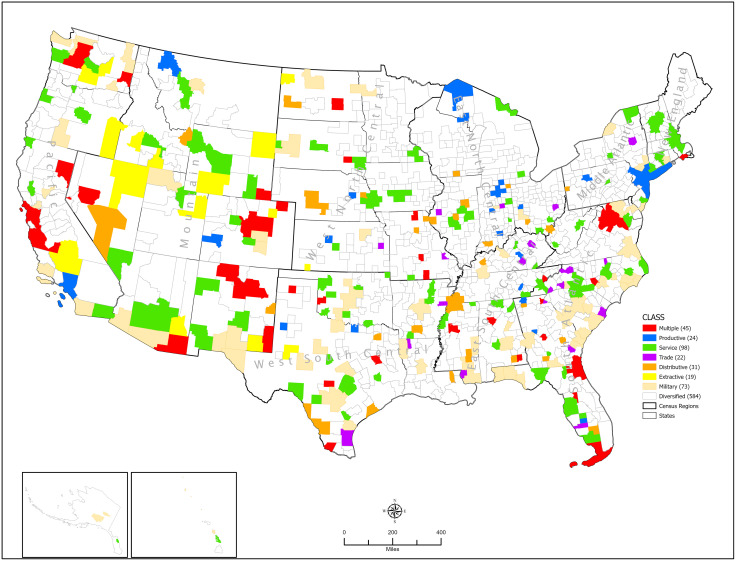
Functional Classification of Metropolitan and Micropolitan Areas, 2021.

Geographically, the areas with multiple specializations were widely dispersed across the country. One of the metro areas with three specialties was located on the East Coast, one on the West Coast, and three were in the central region: Colorado, New Mexico, and Oklahoma. The remaining thirty-eight areas with two functional specialties were scattered across the country. There was a hole in the East North Central and the Middle Atlantic regions. So, in some sense, the distribution was in a “crescent” shape. (Red in Fig 4.)

### 3.4. Single specialty classes

#### 3.4.1. Extractive class.

Extractive industries include growing food, greenhouses, raising livestock, fishing and hunting, forestry and logging, mining, and oil and gas extraction. Nineteen metro/micro areas were determined to have an extractive specialization. To be classified as having a specialty in this industry, the location quotient had to be greater than or equal to the upper outer fence of 9.66. Carlsbad, New Mexico, had the highest location quotient of this group at 16.37, with 21.44 percent of its total employment. Recall that this means Carlsbad had just over sixteen times the national metro/micro area proportion. Carlsbad is heavily involved in oil, gas, and other mineral extraction. In second place was Moses Lake Washington, with 15.69, focused on agriculture. In third was Gillette, Wyoming, at 15.40, centered around coal and oil and gas extraction. While not one of the highest location quotients, Bakersfield, California, is located at the southern end of the fertile San Joaquin Valley and was focused on agriculture.

This Class’s nineteen Metro/Micro areas had a median COS of 0.242. They had a range of 0.2056, from a minimum of 0.154 to a maximum of 0.360. Liberal, Kansas (beef lots, grain, and oil and gas extraction) had the maximum COS, while Bakersfield, California, had the minimum COS.

Interestingly, all areas specialized in agriculture or mining were in the country’s western half. (Yellow in Fig 4.) A listing of the Metropolitan and Micropolitan areas in this class can be found in Table A- 1 in S1 Appendix Tables.

#### 3.4.2. Military class.

The industrial category of the military is problematic for several reasons. One is that the military has small employment numbers in many places. The upper outer fence was only 1.38. Seventy-three areas showed specialization. For some metro/micro areas, the military presence was significant and dominant. Jacksonville, North Carolina, had a location quotient of 45.31 and had 38.12% of its total employment in the military. In second was Hinesville, Georgia, at 33.14. Third was Fairbanks, Alaska, with a location quotient of 22.89. Lawton, Oklahoma, had a location quotient of 21.46.

The seventy-three Metro/Micro areas of the Military Class had a median COS of 0.139, the lowest of the eight Classes. They were the least divergent from the national distribution. They had a minimum COS of 0.051 and a maximum COS of 0.385 for a range of 0.3345, the most extensive spread. Jacksonville, North Carolina, had the maximum COS, while Columbia, South Carolina, had the minimum COS.

The areas specialized in the military were scattered around the country, though there was noticeable clustering in the states along the Atlantic and Gulf coasts. The state of Washington also had a cluster. (Light brown on Fig 4) A listing of the Metro/Micro areas in this class can be found in Table A- 1 in S1 Appendix Tables.

#### 3.4.3. Productive class.

The Productive Class, consisting of Metro/Micro Areas with manufacturing, Information, or construction specializations, garnered just over 18% of the total U.S. metro/micro area employment. It ranged from a low of 5.8% (Wauchula, Florida) to a high of 48% (Kendallville, Indiana). Areas of the Productive Class had the highest median COS of 0.260. They ranged between a minimum of 0.062 and a maximum of 0.338 for a range of 0.2756. Kendallville, Indiana, had the maximum COS, while Los Angeles, California, had the minimum COS. (Blue in Fig 4.) A listing of the Metro/Micro areas in this class can be found in Table A- 2 in S1 Appendix Tables.

***Manufacturing Centers (MFG).*** To be classed as having a specialty in Manufacturing, an area had to have a location quotient over an upper inner fence of 3.34. There were thirteen metro/micro areas with this specialty. Topping the list was Kendallville, Indiana, with a location quotient of 4.37, meaning its employment in Manufacturing (42.8%) was over four times the national metro/micro area proportion. Dalton, Georgia, was second at 3.94 and focused on floor coverings and carpeting. Sidney, Ohio, was third at 3.90 and is focused on automobile engines and related components. No metro/micro area exceeded the location quotient for the upper outer fence of 4.89. The Manufacturing Center of Kendallville, Indiana, had the maximum COS of 0.335, and Dayton, Tennessee, had the lowest COS (0.248) of the manufacturing centers.

All but two of the manufacturing centers were located on a north-south axis in five states: Michigan, Indiana, Ohio, Tennessee, and Georgia. One of the remaining two was in Pennsylvania, and the second was in Nebraska.

***Information Centers (INF).*** The information category is sector 51, which includes print and software publishing, motion pictures, radio and television broadcasting, data processing, and telecommunications. The cut-off point for an area classed as an information center was 1.60. Ten metro/micro areas qualified for this designation. The area with the highest LQ was Danville, Kentucky, at 2.68 (5.05% of its total employment). In second was Hayes, Kansas, at 2.11, and Levelland, Texas was third with 2.08. Only one area exceeded the upper outer fence of 2.338, which was Danville, Kentucky. The COS for Danville, Kentucky, was 0.2002; for Hayes, Kansas, it was 0.1827; and for Levelland, Texas, it was 0.1938.

Information centers were geographically scattered throughout the country, though Texas did have two.

***Construction Centers (CON).*** The construction industry is a category that, like the military, is endemic. This category involves activities involved with the actual construction or building something [14]. The upper outer fence was 2.60. Only two areas specialized in construction. Arcadia, Florida, had a location quotient of 3.28 with 22.16% of its total employment, and Thomaston, Georgia, had a quotient of 2.87, with 19.37%. The COS for Arcadia, Florida, was 0.3379, while for Thomaston, Georgia, it was 0.2179. Both areas were in the southeast of the country.

#### 3.4.4. Distributive class.

The Distributive Class, consisting of Metro/Micro Areas specializing in transportation, warehousing, utilities, and wholesaling, garnered just over 9% of all Metro/Mico employment. Laramie, Wyoming, had the lowest proportion (0.0%), while Elk City, Oklahoma, had the highest (26.2%). Metro areas of this class had a median COS of 0.196. They ranged between a minimum of 0.097 and a maximum of 0.332 for a range of 0.2353. Jasper, Indiana, had the maximum COS, while Memphis, Tennessee, had the minimum COS. A listing of the Metro/Micro areas in this class can be found in Table A- 3 in S1 Appendix Tables.

***Transportation, Warehousing, and Utilities Centers (TWU).*** For an area to specialize in transportation, warehousing, and utilities, its location quotient had to be greater than or equal to 1.77. Three metro/micro areas exceeded the upper outer fence of 2.39: Laredo, Texas; North Platte, Nebraska; and Pahrump, Nevada. There were twenty-one centers. North Platte, Nebraska, had an LQ of 2.96, the location of a huge railroad yard. In second was Pahrump, Nevada, at 2.88. Laredo, Texas, was third with an LQ of 2.46, which is the site of a large inland port and an extensive border crossing. Memphis, Tennessee, had an LQ of 2.34 and is the location for an extensive FedEx distribution center. Lexington, Nebraska, had the highest TCU COS (0.308), while Memphis, Tennessee (0.097), had the lowest.

***Wholesale Centers (WHO).*** Wholesale trade (sector 42) are establishments that sell goods for resale to other establishments for sale or as an intermediate material for Manufacturing. They are intermediary points. To be classified as a wholesale center, a metro/micro area had to have a location quotient greater than or equal to 2.05. There were fifteen areas specialized in wholesaling. Sulphur Springs, Texas, was highest, with an LQ of 3.31. Moultrie, Georgia, had the second highest at 3.03, and the third was Angola, Indiana, at 2.87. Three areas exceeded the upper outer fence of 2.925: Sulfur Springs, Texas; Moultrie, Georgia; and Jasper, Indiana. Geographically, there was no pattern in the location of wholesale centers other than in manufacturing and agricultural areas. Jasper, Indiana, had the highest wholesale center COS (0.332), and New Castle, Pennsylvania, had the lowest (0.109).

Geographically, this Class did not display any strong pattern. Metro/Micro Areas specialized in distribution were centered more in the East North Central region. (Orange on Fig 4.)

#### 3.4.5. Trade class.

Trade centers included areas that specialized in retail trade. Retail trade establishments (sectors 43 and 44) sell goods directly to the general public.

Retail centers had a location quotient greater than or equal to 1.59. Retail trade is endemic to both urban areas and rural communities. For all metro/micro areas, the national percentage employed in retail trade was just under eleven percent. Kingsville, Texas, was highest, with an LQ of 2.16. In second was Boone, North Carolina, at 1.97, and third was Macomb, Illinois, at 1.91. Only Kingsville, Texas, exceeded the upper outer fence of 1.988. The median COS for the Trade Class was 0.192. The range was 0.136 between the minimum of 0.107 in Picayune, Mississippi, and the maximum range of 0.242 in Forest City, Arkansas.

Retail trade centers were all located in the eastern half of the country. An interesting concentration was in the Appalachian Mountains of North Carolina and Georgia. (Purple in Fig 4.) A listing of the Metro/Micro areas in this class can be found in Table A- 4 in S1 Appendix Tables.

#### 3.4.6. Service class.

This category includes all industries that assist or service businesses and private citizens in some way. Here are finance and insurance, real estate, professional and technical services, management companies, educational services, health services, construction, and public administration. The U.S. economy’s being service-oriented was demonstrated by the fact that in 2021, just over 60% of the total metro/micro employment was employed in service functions. (Green in Fig 4.) The median Service Class employment of the metro/Micro areas was 56.12%. The lowest proportion was 30.6%, which was found in Jasper, Indiana. The highest proportion was 74.6% and was found in Laramie, Wyoming. A listing of the Metro/Micro areas in this class can be found in Table A- 5 in S1 Appendix Tables.

This Class’s ninety-four metro/Micro Areas had a median COS of 0.193. The maximum COS was 0.333 in Beeville, Texas, while the minimum COS was in Omaha, Nebraska, at 0.052. The range was 0.2862.

***FIRE Centers.*** For a metro/micro area to be classified as having a specialty in FIRE, it had to have a location quotient greater than or equal to 1.43. There were twelve areas. Bloomington, Illinois, was the highest, with an LQ of 2.86 and a COS of 0.1609. In second place was Des Moines, Iowa, at 2.13 and a COS of 0.0921; in third was Sioux Falls, South Dakota, at 1.77 and a COS of 0.0983. These three are large insurance centers, as are Omaha, Nebraska, and Hartford, Connecticut. Two areas exceeded the upper outer fence of 1.993: Bloomington, Illinois, and Des Moines, Iowa.

***Professional, Scientific, and Management Centers (PRO).*** This industry category was often found to be one of the specialties of the multiple specialty metro/micro areas. To be classified as having this as a specialty, a metro/micro area had to have a location quotient of 1.30. Six areas had this functional specialty. The highest was Wauchula, Florida, with an LQ of 1.92 with a COS of 0.2766. Easton, Maryland, followed in second at 1.69 with a COS of 0.1369. Raleigh, North Carolina, was third at 1.59 and COS of 0.9528. Wauchula, Florida, exceeded the upper outer fence of 1.790.

***Education and Health Services Centers (E & H).*** Educational services include all educational facilities, elementary, secondary, high school, and college. Health services include hospitals, nursing homes, doctors, and medical laboratories. Both industrial groups are endemic to communities. To obtain a level of a specialty, a metro/micro area had to have a location quotient of 1.53. There were fifteen metro/micro areas with this specialty. Most of the items on this list were not too surprising. Ithica New York (Cornell University) was in first place at 2.00 with a COS of 0.2515. In second place was Rocester, Minnesota (home of the Mayo Clinic) at 1.84 and a COS of 0.2028; in third was Silver City, New Mexico, with a COS of 0.2813. Others include Stillwater, Oklahoma (Oklahoma State University), and Corvallis, Oregon (Oregon State University). Ithaca, New York, was the only area to exceed the upper outer fence of 1.91.

***Leisure and Hospitality Centers (L & H).*** This combined category comprises two sectors, sector 71 (arts, entertainment, and recreation) and sector 72 (accommodation and food services). Seventeen metro/micro areas had a location quotient greater than or equal to the cut-off of 1.75. First was Hailey, Idaho (resorts, especially skiing), with an LQ of 3.14 and a COS of 0.2714. In second was Kahului, Hawaii, at 2.95 with a COS of 0.1914; in third was Edwards, Colorado (skiing, near Vail) at 2.73 with a COS of 0.2832. Branson, Missouri, had this specialty, as did Las Vegas, Nevada. Exceeding the upper outer fence of 2.301 were Hailey, Idaho, Edwards; Colorado, Kahului, Hawaii; Kill Devil Hills, North Carolina; Las Vegas, Nevada; Newport, Oregon; and Ruidoso, New Mexico.

***Other Services Centers (OTH).*** This category is a catch-all. An industry not elsewhere classified is found here. The category includes repair and maintenance, private households, personal services, religious organizations, and professional organizations. Twenty-four areas exceeded the cut-off point of 1.70. Uvalde, Texas, was the highest, with an LQ of 2.79 and COS of 0.3090. Rockingham, North Carolina, was second with 2.34 and a COS of 0.1883; Weatherford, Oklahoma, was third with 2.23 and 0.1981. Exceeding the upper outer fence were Uvalde, Texas; Rockingham, North Carolina; and Weatherford, Oklahoma.

***Public Administration Centers (PUB).*** Besides the typical employment of government agencies, this category includes tribal governments and correctional institutions. The cut-off point was a location quotient of 2.28. Twenty-four areas had a specialty in this category. Juneau, Alaska, had the highest at 5.08 and the COS at 0.2779. Second place was Del Rio, Texas, at 4.08 and a COS of 0.2475. In third was Altus, Oklahoma, at 3.92 and a COS of 0.2699. Altus, Oklahoma; Beeville, Texas; Del Rio, Texas; Helena, Montana; Juneau, Alaska; and Pierre, South Dakota, exceeded the upper outer fence. Several state capitals were on the list, some of which were Springfield, Illinois; Pierre, South Dakota; Helena, Montana; and Tallahassee, Florida.

#### 3.4.7. Diversified class.

Any metro/micro area that did not exceed one of the cut-off points for any classification category was classified as diversified. This large class contained 584 metro/micro areas (65.3% of all metro/micro areas). (No color in Fig 4.)

The Diversified Class had a median COS of 0.144. Nashville, Tennessee, had the minimum COS (0.031), and Huron, South Dakota, had the maximum COS (0.298). The range was 0.2677. Being classified as diversified does not mean that a metro or micro area lacks a concentration of employment in any given industry. It simply was not high enough to breach the cutoff point. The diversified class was not a totally homogenized mass. For example, Celina, Ohio, had a manufacturing location quotient of 3.31, but it was below the cutoff of 3.34. Another example was Keene, New Hampshire, which had a wholesale trade location quotient of 2.04 but was below the cutoff point of 2.05. A third example was Odessa, Texas, which had an extractive location quotient of 9.25 but was below the cutoff of 9.66. Chicago, Illinois; Dallas, Texas; Houston, Texas; and Philadelphia, Pennsylvania were among the areas classified as diversified.

As shown above, the cutoff points are very sharp – the lines are in fact fuzzy. To examine the areas that are close to cutoff points — those areas that were “close but not quite there”, the following process was employed. For extractive and construction functions, areas with location quotients that were between the lower outer fence and the upper outer fence were selected. For all other functions, those areas that had a location quotient within ten percent of the lower fence were picked. The choice of ten percent was admittedly arbitrary. A total of 153 metro areas fell into this group.

There were thirteen Diversified Metro/Micro Areas that had an orientation to the Extractive Class functions, falling between the upper inner fence (LQ = 6.36) and the upper outer fence (LQ = 9.66). Some were just under the cutoff threshold point for selection as a functional specialty. Odessa, Texas, had an Extractive LQ of 9.25, just short of the threshold. Next was Alice, Texas, with an LQ of 8.90. Both were centers for oil and gas extraction. Third was Wenatchee, Washington, with an LQ of 8.72, specializing in agriculture, mainly apples. This group had a Coefficient of Specialization that ranged from a low of 0.12 in Fresno, California, to a high of 0.30 in Huron, South Dakota.

Geographically, the diversified metro/micro areas that had some sub-dominance in extractive functions were all west of the Mississippi River. The Pacific region had five, the West North Central region had five, and the West South Central region had four. This pattern matches that of the Metro/Micro Areas that were in the Extractive Class.

Retail trade functions showed a large number of metro areas within ten percent of the upper inner fence (LQ between 1.43 and 1.59). Twenty-four areas were identified as having a moderate dominance in retail trade. At the top of the list was Fayetteville-Springdale, Arkansas, with a LQ of 1.57, which is the home base for Walmart. Dyersburg, Tennessee, was next with a LQ of 1.56, and the third was Eau Clair, Wisconsin, with a LQ of 1.55. Geographically, seven were in the West South Central Region, and seven were in the East South Central Region. None were located in the Mountain or the Pacific Regions. Of this group, Plainville, Texas, had the highest Coefficient of Specialization (COS) of 0.255, while Rutland, Vermont, had the lowest at 0.12 (Table 3).

**Table 3:** Diversified Class Areas With a Sub-dominant Function.

FUNCTIONAL CLASS		Lower Limit	Upper Limit	Number of Areas
**MILITARY**	Military	0.86 (upper inner fence)	1.38 (upper outer fence)	20
**EXTRACTIVE**	Agriculture, forestry, fishing and hunting, and mining	6.36 (upper inner fence)	9.66 (upper outer fence)	13
**PRODUCTIVE**				
	Manufacturing	3.006	3.34	11
	Information	1.44	1.60	8
	Construction	1.93 (upper inner fence)	2.60 (upper outer fence)	7
**DISTRIBUTIVE**				
	Transportation and warehousing, and utilities	1.593	1.77	9
	Wholesale trade	1.845	2.05	8
**TRADE**	Retail Trade	1.431	1.59	25
**SERVICES**				
	Finance and insurance, and real estate and rental and leasing	1.287	1.43	15
	Professional, scientific, and management, and administrative and waste management services	1.17	1.30	12
	Educational services, and health care and social assistance	1.377	1.53	0
	Leisure & Hospitality	1.575	1.75	23
	Other services, except public administration	1.53	1.70	21
	Public administration	2.052	2.25	8
TOTAL				153

Twenty-three metro areas fell into this zone of possible moderate dominance in Productive functions (manufacturing, information, and construction) within a ten percent range (for manufacturing, between 3.006 and 3.34; for information, between 1.44 and 1.60, and for construction, between 1.93 and 2.60). For manufacturing, Celina, Ohio, tops the list with a LQ of 3.31, followed by Bennettsville, South Carolina, with a LQ of 3.24, and in third place is Manitowoc, Wisconsin, at 3.23. The north-south axis was also identified in this group, although it extended to the northwest, encompassing Iowa, Wisconsin, and Minnesota. Of the thirteen metro/micro areas, 54% were in the East North Central census region, with six being in Indiana, Ohio, and along the southern border of Michigan. Four metro areas had a sub-dominance in information, three of which were in the Middle Atlantic and New England Regions. Texas had the remaining area. There were seven areas with moderate dominance in the construction sector. Topping this list was Taos, New Mexico, with a LQ of 2.56. The second was Okeechobee, Florida, at 2:31. These metro areas were scattered throughout the county, though none were located in the Pacific Region. The Coefficient of Specialization (COS) for these areas ranged from a low of 0.08 in Burlington, Vermont, to a high of 0.30 in Storm Lake, Iowa.

Interestingly, twenty-four metro areas had possible multiple sub-dominant specializations. Two of the areas had three. Steamboat Springs, Colorado, had a dominance in construction, FIRE, and other services. Stephenville, Texas, had a dominance in agriculture, retail trade, and information. Pairings of functions showed no consistency. Geographically, the Pacific Region had six of these metro areas, the Mountain Region had six, and the West South Central Region had five. The Coefficient of Specialization (COS) ranged between a low of 0.09 in Santa Cruz, California, to a high of 0.20 in Greenville, Mississippi.

In all cases other than these twenty-four, if a Metro/Micro area’s economy contained an industrial type that was moderately dominant, there was only one dominant.

## 4. Discussion & summary

**Fig 5:**
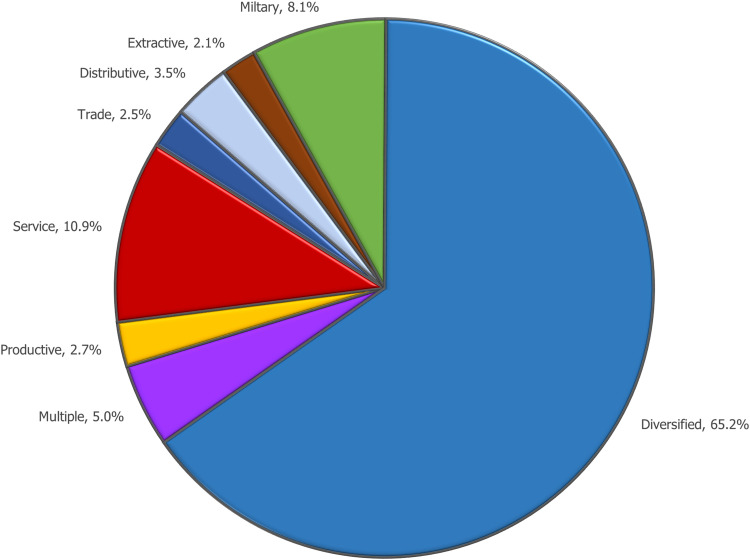
Metropolitan and Micropolitan Area Employment Centers by Functional Class, 2021.

Most (66%) of the Metro/Micro Areas were of the Diversified Class. None of the 584 metro/Micro Areas in this class had employment in a category that exceeded a cut-off point for a specialization designation. As seen in Fig 5, the second largest class of metro areas was the Service Class, with 11%. In third place were those areas in the Military Class. Metro areas that did have a specialty were dominated by only one functional category (28.4%). There were only a few metro areas that had multiple specializations. Of the 927 Metropolitan and Micropolitan areas, only forty-five areas (5.0%) had two or more specializations. Only five (just over half a percent) had three specializations: Elk City, OK; Espanola, NM; Seattle, WA; Sterling, CO; and Washington, DC.

**Fig 6:**
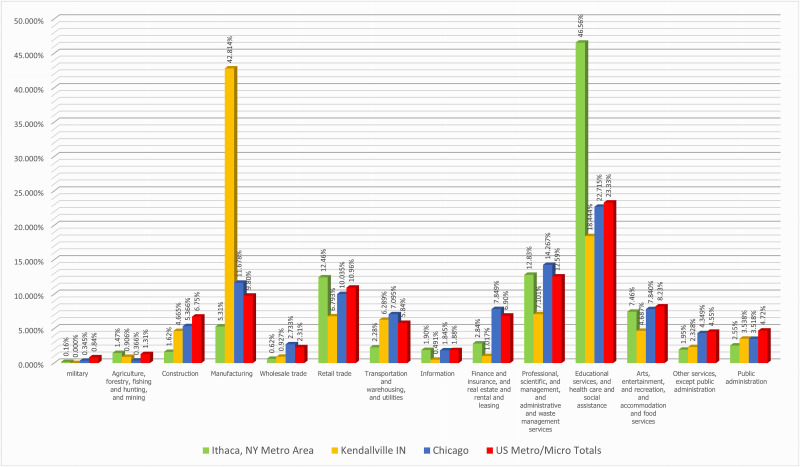
Comparison Between a Diversified MA, A Service Class MA, A Productive Class MA, and the U.S. Metro-Micro Totals, 2021.

In general and unsurprisingly, the structures of the metro/micro area economies were found to be different, and Fig 6 illustrates this graphically. This is a comparison of a Diversified Class metro area (Chicago, Illinois), a Productive Class metro area specializing in Manufacturing (Kendallville, Indiana), and a Service Class Metro area specialized in Education and Health Services (Ithaca, New York), against the totals for all metro/micro areas.

Kendallville had the highest percentage of its total employment in a single category (Manufacturing, 42.8%). Its location quotient was 4.4, or over four times the national proportion. No other industrial category for Kendallville was above the national proportions. In comparison, Chicago had 11.7% of its total employment in manufacturing, with a location quotient of 1.19. Ithaca only had 5.3% of its total employment in manufacturing for a location quotient of only 0.54. Kendallville and Ithaca also differed significantly in the retail and professional functions, where Ithaca had higher proportions but was still lower than national levels.

For a second comparison, Fig 6 also shows that Ithaca, New York, which specialized in education and health functions, had 46.6% employed in E&H for a location quotient of 2.0, which was the highest for any metro/micro area on E&H (the cut-off was 1.53). The graph shows that no other industrial category stands out in Ithaca. The only other industrial group slightly higher than the national Metro/Micro area proportion was retail trade, 12.46% versus 10.96%. In contrast, Kendallville had 18.4% employed in E&H with a location quotient of only 0.79.

**Fig 7:**
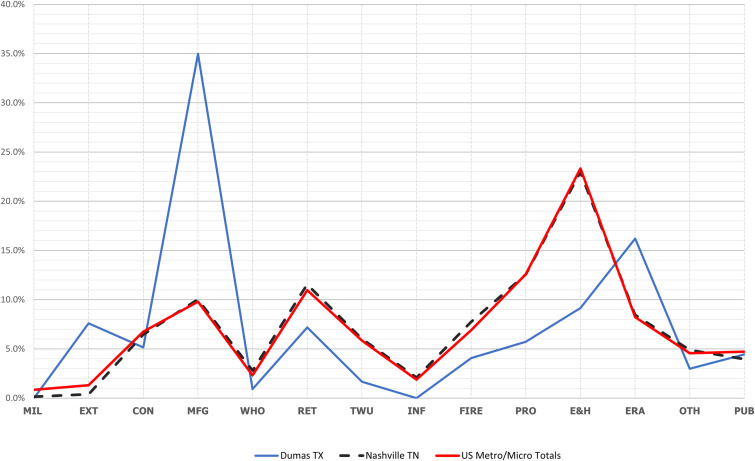
Comparison Between Nashville, TN, That had the Lowest COS; Dumas, TX, That had the Highest and the U.S. Metro-Micro Totals, 2021.

The Coefficient of Specialization (COS) highlights the difference between an MA profile and a national one (Fig 7). The metro/micro areas with specializations deviated most from the national employment structure. The twenty-four areas in the Productive Class displayed the widest divergence from the national distribution. They had a median COS of 0.26. This means that 26% of those employed must shift to other employment categories to match the national distribution structure. The Extractive Class of metro/micro areas had the second-highest median COS of 0.242 (24%). The Class with the lowest COS was the Military Class at 0.14 (14%).

The Multiple Class had a COS of 0.22 (22%), but it contained the metro/micro area with the highest COS (0.39). The 584 metro areas in the Diversified Class, i.e., those with no functional specialization, had a median COS of 0.144. These also had the lowest COS at 0.031.

Table 4 visualizes the extremes for COS: the metro area with the highest, the metro area with the lowest, and the national distribution. The metro area with the lowest COS (0.0307 or 3.1%) was Nashville, Tennessee, classified as being in the Diversified Class. Only three percent of Nashville’s total employment would need to shift to match the national distribution. The graph shows that the employment distribution had little deviation from the national distribution. Its greatest, but minor, deviation was for military and extractive functions.

**Table 4:** Coefficient of Specialization (COS) by Functional Class, 2021.

CLASS	Orientation	Count	Median	Minimum	Maximum	Range
Distributive		31	0.1960	0.0971	0.3324	0.2353
Diversified	All	584	0.1441	0.0307	0.2984	0.2677
	Extractive	13	0.2265	0.1185	0.2844	0.1799
	Manufacturing	12	0.2500	0.2292	0.2971	0.0741
	Multiple	23	0.1948	0.0900	0.2534	0.1635
Extractive		19	0.2418	0.1545	0.3601	0.2056
Military		73	0.1392	0.0507	0.3852	0.3345
Multiple		45	0.2207	0.0877	0.3941	0.3064
Productive		24	0.2599	0.0622	0.3379	0.2757
Services		94	0.1927	0.0518	0.3373	0.2820
Trade		22	0.1925	0.1069	0.2425	0.1356

In sharp contrast was the profile of Dumas, Texas, which had a COS of 0.394, indicating that 39.4% of its total employment would need to shift to match the national distribution. Dumas was classed as being in the Multiple Class. The graph shows that Dumas deviates from the national distribution for all categories. It significantly exceeded the national proportions for the extractive, manufacturing, and entertainment functions. For all other functions, it was lower than the national proportions.

The Metro/Micro Areas in the Productive Class displayed the most divergence from the national employment distribution with a median Index of Divergence of 0.260. The twenty-four areas in the Productive class would have to shift 26% of their employment to different categories to match the national pattern. The Extractive Class, with nineteen areas, had the second-highest index at 0.242. The Multiple Class, containing forty-three areas, had the third highest index at 0.224.

In conclusion:

1. There was typically only one if a Metro/Micro area had a functional specialization.
2. Only forty-five areas (5.0%) had two or three specializations, with only five having three specializations.
3. Most Metro/Micro areas (584 or 67%) were found to have no specialization; they were classed as diversified.
4. The Service Class was second most numerous (10.5%). The Military Class metro/Micro areas were the third most common.
5. Metro/Micro areas with a functional specialization deviated most from the national industrial pattern.
6. Metro areas of the Productive Class had the most significant deviation from the national pattern, with just under 26% of employment having to change categories to match the national pattern. The Extractive Class followed in second.
7. The military Class was the least divergent from the national pattern.

Please note that this functional classification, as with any classification, does not preclude other types of classifications. It does not preclude, for example, New York City being characterized as the performing arts center of the country or being recognized as a financial center. Nor does it preclude Pittsburg from still being a significant industrial center for steel production.

Further research directions lie in utilizing this classification are numerous. Below are four possible avenues.

1. One is the relationships with the social environment. A metro area’s class does not in itself have a direct effect on the socio-cultural environment. It is indirect. So, the first step would be to determine the effects of the functional class on intermediary socio-demographic variables. Included here would be:
  a. Sex ratio
  b. Dependency ratios (both senior and minor)
  c. Household income
  d. Occupational composition
  e. Educational attainment and enrollment
  f. Racial and ethnic diversity
  g. Racial and ethnic segregation
  h. Crime rates and drug use.

Intriguing future research questions could include:

- Are the Metro/Micro areas that are highly specialized in, say, the Production Class, such as Kendallville, Indiana (manufacturing), socio-culturally structured differently than, say, Metro/Micro areas that are highly specialized in the Services Class, such as Ithaca, New York (education and health services)?
- Are the Metro/Micro areas that are highly specialized in, say, the Production Class more racially segregated than, say, Metro/Micro areas that are highly specialized in the Services Class?
- Are the metro/Micro areas of the Multiple Class significantly different from those of the Productive Class on the socio-demographic variables such as the sex ratio or, the senior dependency ratio?
  2. Another avenue for future research is the relationship between functional classes and the characteristics of their housing markets. Variables could include tenure, age of structures, structure types, housing conditions, housing values, and rents. Research questions such as these could be covered:
- Are there significant differences in the types of housing structures between Metro/Micro areas of the Production Class and those of Metro/Micro areas of the Service Class?
- In the tenure mix?
- In the age of the housing stock?
- In housing values and rents?
  3. A third interesting research direction could be to explore if the age of the Metro/Micro area is related to the functional class. Have older metro/micro areas tended to be more of one functional class over the other classes? Or do newer metro/areas tend to be more of one functional class over others? Defining the age of a metropolitan area is a challenging task. Measuring the age could be based on the percentage of its housing stock built before 1939, the date its primary city was incorporated, or the date the Metro/Micro area was first designated as a metropolitan area.
  4. A fourth area of exploration would be to do a time series, tracking changes in the functional classifications over time.

This avenue presents significant issues and impediments that must be addressed in some way. One issue is that the number of metro and micro areas changes over time. Metro areas are added and dropped approximately every ten years; an area classified as a metro area in 2020 may not have been classified as such in 2010 (and vice versa). For example, Abilene, Texas, was added in 1960; Ada, Oklahoma, was added in 2003; Anderson, South Carolina, was added in 1981; Dillon, South Carolina, was removed in 2023; and Harrisburg, Illinois, was removed in 2013 [32].

These changes are in addition to any ripple effects from changes in the definition of urban (recently revised from a place with a population of 2,500 to one with a population of 5,000) [6,33] and from any changes in the definition of urbanized areas, as well as the constant physical expansion (or shrinkage) of the urbanized area’s physical footprint.

A second and more troubling issue is that the physical area (and thus population and employment) of the area changes between censuses due to counties being added or dropped. The Chicago Metro Area is a good example: from the original six counties in 1950, seven were added, four were acquired through transfer from another area, and two were deleted through transfer to a different area. Cincinnati, Ohio, is another good example: The original in 1950 had three counties, and over the years, one was deleted, and fourteen were added [32].

The third set of impediments pertains to changes in the definitions of both employment and occupation categories. These have relatively minor changes every couple of years. However, there were significant changes in the definitions of occupations in the 1950s and again in the 1970s. The Census Bureau issues warnings about comparing occupation data across time [25]. The conversion of the industry classification system from the Standard Industrial Classification (SIC) to the North American Industrial Classification System (NAICS) in the 1990s produced issues with comparability between time periods. These changes make it very difficult, if not nearly impossible, to go back in time for comparisons.

Going forward in time, a time series would not encounter major problems associated with changes in industry and occupation definitions. However, issues with changes in the units of analysis will remain.

## Supporting information

S1 Appendix Tables. (DOCX)

S2 Appendix NAICS Employment Categories. (DOCX)
