# Supplementary material for: A new functional classification of U.S. metropolitan and micropolitan areas
Source: PLoS One. 2025 Oct 17;20(10):e0334284. doi: 10.1371/journal.pone.0334284 (PMC12533906; doi:10.1371/journal.pone.0334284)
Supplement: S1 Appendix — (DOCX) [file pone.0334284.s001.docx]

# **S1 Appendix Tables**

Note: Areas in bold have the highest location quotient in their respective functional class.

A- 1 Metropolitan and Micropolitan Areas Specializing in Extractive Functions, 2021

| **Agriculture & Mining** | **Military** | |
| --- | --- | --- |
| Bakersfield, CA | Abilene, TX | Manhattan, KS |
| Big Spring, TX | Alamogordo, NM | Meridian, MS |
| Burley, ID | Alexandria, LA | Minot, ND |
| **Carlsbad-Artesia, NM (LQ = 16.37)** | Anchorage, AK | Montgomery, AL |
| Elko, NV | Augusta-Richmond County, GA-SC | Morehead City, NC |
| Fort Morgan, CO | Baltimore-Columbia-Towson, MD | Mount Vernon-Anacortes, WA |
| Gillette, WY | Bremerton-Silverdale-Port Orchard, WA | New Bern, NC |
| Liberal, KS | Charleston-North Charleston, SC | Norwich-New London, CT |
| Midland, TX | Clarksville, TN-KY | Oak Harbor, WA |
| Moses Lake, WA | Colorado Springs, CO | Ocean City, NJ |
| Mountain Home, ID | Columbia, SC | Ogden-Clearfield, UT |
| Ontario, OR-ID | Columbus, GA-AL | Oklahoma City, OK |
| Price, UT | Corpus Christi, TX | Panama City, FL |
| Rock Springs, WY | Crestview-Fort Walton Beach-Destin, FL | Pensacola-Ferry Pass-Brent, FL |
| Safford, AZ | Dayton-Kettering, OH | Port Angeles, WA |
| Vernal, UT | Dover, DE | Poughkeepsie-Newburgh-Middletown, NY |
| Visalia, CA | Elizabethtown-Fort Knox, KY | Pueblo, CO |
| Williston, ND | El Paso, TX | Rapid City, SD |
| Yakima, WA | Enid, OK | San Angelo, TX |
|  | Fairbanks, AK | San Antonio-New Braunfels, TX |
|  | Fayetteville, NC | San Diego-Chula Vista-Carlsbad, CA |
|  | Goldsboro, NC | Santa Maria-Santa Barbara, CA |
|  | Grand Forks, ND-MN | Savannah, GA |
|  | Great Falls, MT | Shelby, NC |
|  | Gulfport-Biloxi, MS | Shreveport-Bossier City, LA |
|  | Hattiesburg, MS | Spokane-Spokane Valley, WA |
|  | Hilton Head Island-Bluffton, SC | Sumter, SC |
|  | Hinesville, GA | Talladega-Sylacauga, AL |
|  | **Jacksonville, NC (LQ = 45.31)** | Tucson, AZ |
|  | Killeen-Temple, TX | Urban Honolulu, HI |
|  | Klamath Falls, OR | Valdosta, GA |
|  | Kokomo, IN | Vallejo, CA |
|  | Las Cruces, NM | Virginia Beach-Norfolk-Newport News, VA-NC |
|  | Lawton, OK | Walla Walla, WA |
|  | Little Rock-North Little Rock-Conway, AR | Watertown-Fort Drum, NY |
|  |  | Wichita Falls, TX |
|  |  | Yuba City, CA |
|  |  | Yuma, AZ |

A- 2 Metropolitan and Micropolitan Areas Specializing in Productive Functions, 2021

| **Manufacturing** | **Information** | **Construction** |
| --- | --- | --- |
| Columbus, NE | **Danville, KY (LQ = 2.68)** | **Arcadia, FL (LQ = 3.28)** |
| Dalton, GA | Gainesville, TX | Thomaston, GA |
| Dayton, TN | Hays, KS |  |
| Elkhart-Goshen, IN | Houghton, MI |  |
| Frankfort, IN | Iron Mountain, MI-WI |  |
| **Kendallville, IN (LQ = 4.37)** | Kalispell, MT |  |
| Plymouth, IN | Levelland, TX |  |
| St. Marys, PA | Los Angeles-Long Beach-Anaheim, CA |  |
| Seymour, IN | Montrose, CO |  |
| Sidney, OH | New York-Newark-Jersey City, NY-NJ-PA |  |
| Sturgis, MI |  |  |
| Warsaw, IN |  |  |

A- 3 Metropolitan and Micropolitan Areas Specializing in Distributive Functions, 2021

| **Transportation, warehousing & Utilities** | **Wholesale Trade Centers** |
| --- | --- |
| Camden, AR | Angola, IN |
| Clewiston, FL | Bay City, TX |
| Eagle Pass, TX | Calhoun, GA |
| Enterprise, AL | Clovis, NM |
| Hammond, LA | Dickinson, ND |
| Laredo, TX | Fremont, NE |
| Laurel, MS | Janesville-Beloit, WI |
| Lexington, NE | Jasper, IN |
| Madisonville, KY | Lincoln, IL |
| Memphis, TN-MS-AR | Moultrie, GA |
| Moberly, MO | New Castle, PA |
| **North Platte, NE (LQ = 2.96)** | Pontiac, IL |
| Pahrump, NV | Quincy, IL-MO |
| Point Pleasant, WV-OH | Rexburg, ID |
| Vidalia, GA | **Sulphur Springs, TX (LQ = 3.31)** |
| Wilmington, OH |  |

A- 4 Metropolitan and Micropolitan Areas Specializing in Retail Trade Functions, 2021

| **Retail Centers** | |
| --- | --- |
| Amsterdam, NY | **Kingsville, TX (LQ = 2.16)** |
| Boone, NC | Macomb, IL |
| Brevard, NC | Marion, IN |
| Cambridge, OH | Minden, LA |
| Farmington, MO | Mount Sterling, KY |
| Forest City, NC | North Wilkesboro, NC |
| Forrest City, AR | Ottawa, KS |
| Georgetown, SC | Picayune, MS |
| Gloversville, NY | Punta Gorda, FL |
| Henderson, NC | Somerset, KY |
| Jesup, GA | Toccoa, GA |

A- 5 Metropolitan and Micropolitan Areas Specializing in Service Functions, 2021

| **FIRE** | **Professional** | | **Education & Health** |
| --- | --- | --- | --- |
| **Bloomington, IL (LQ = 2.86)** | Austin-Round Rock-Georgetown, TX | Ann Arbor, MI | |
| Bridgeport-Stamford-Norwalk, CT | Boston-Cambridge-Newton, MA-NH Area | Athens, OH | |
| Charlotte-Concord-Gastonia, NC-SC | Easton, MD | Champaign-Urbana, IL | |
| Des Moines-West Des Moines, IA | Huntsville, AL | Clarksdale, MS | |
| Hartford-East Hartford-Middletown, CT | Raleigh-Cary, NC | Corvallis, OR | |
| Heber, UT | **Wauchula, FL (LQ = 1.92)** | Gallup, NM | |
| Magnolia, AR |  | **Ithaca, NY (LQ = 2.00)** | |
| Naples-Marco Island, FL |  | Laramie, WY | |
| Omaha-Council Bluffs, NE-IA |  | Lebanon, NH-VT | |
| Phoenix-Mesa-Chandler, AZ |  | Lewisburg, PA | |
| Sioux Falls, SD |  | Portsmouth, OH | |
| Tampa-St. Petersburg-Clearwater, FL |  | Rochester, MN | |
|  |  | Silver City, NM | |
|  |  | Stillwater, OK | |
|  |  |  | |
| **Leisure & Hospitality** | **Other Services** | **Public Administration** | |
| Atlantic City-Hammonton, NJ | Batesville, AR | Aberdeen, WA | |
| Branson, MO | Bluffton, IN | Altus, OK | |
| Cullowhee, NC | Brownwood, TX | Beeville, TX | |
| Durant, OK | Bluffton, IN | Cañon City, CO | |
| Edwards, CO | Clearlake, CA | Crescent City, CA | |
| **Hailey,** **ID (LQ = 3.14)** | Cleveland, MS | Del Rio, TX | |
| Hood River, OR | Cleveland, TN | Deming, NM | |
| Jackson, WY-ID | Connersville, IN | El Centro, CA | |
| Kahului-Wailuku-Lahaina, HI | Corinth, MS | Elkins, WV | |
| Kill Devil Hills, NC | Decatur, IN | Frankfort, KY | |
| Las Vegas-Henderson-Paradise, NV | Duncan, OK | Helena, MT | |
| Miami, OK | Fort Payne, AL | Huntsville, TX | |
| Newport, OR | Great Bend, KS | Juneau, AK | |
| Payson, AZ | Rockingham, NC | Lake City, FL | |
| Riverton, WY | Searcy, AR | Malone, NY | |
| Ruidoso, NM | Selma, AL | Middlesborough, KY | |
| Sevierville, TN | Tifton, GA | Mount Gay-Shamrock, WV | |
|  | **Uvalde, TX (LQ = 2.79)** | **Pierre, SD (LQ = 4.24)** | |
|  | Washington, NC | Plattsburgh, NY | |
|  | Weatherford, OK | Prineville, OR | |
|  | Woodward, OK | Shelton, WA | |
|  |  | Springfield, IL | |
|  |  | Tallahassee, FL | |
|  |  | Taylorville, IL | |
