# Supplementary material for: A new functional classification of U.S. metropolitan and micropolitan areas
Source: PLoS One. 2025 Oct 17;20(10):e0334284. doi: 10.1371/journal.pone.0334284 (PMC12533906; doi:10.1371/journal.pone.0334284)
Supplement: S2 Appendix — (DOCX) [file pone.0334284.s002.docx]

**S2 Appendix NAIC Employment Categories**

The U.S. Bureau of the Census follows the conventions set in the North American Industrial Classification System (NAICS). The conceptual framework is constructed around industries that have common production processes. At the highest level, there are twenty sectors. These sectors are then divided into ninety-nine subsectors. These are further subdivided into 311 industrial groups and 709 NAICS industries. (14) For the American Community Survey reporting, the Census Bureau reassembles these categories into thirteen groups, plus the military.

1. Military
2. Agriculture, forestry, Fishing and Hunting, and Mining [herein termed Extractive]
   1. Sector 11. Activities of this sector are growing crops, raising animals, harvesting timber, and harvesting fish and other animals from farms, ranches, or the animals' natural habitats.
   2. Sector 21. Activities of this sector are extracting naturally occurring mineral solids, such as coal and ore; liquid minerals, such as crude petroleum; and gases, such as natural gas; and beneficiating (e.g., crushing, screening, washing, and flotation) and other preparation at the mine site, or as part of mining activity.
3. Construction

Sector 23. Activities of this sector are erecting buildings and other structures (including additions), heavy construction other than buildings, and alterations, reconstruction, installation, maintenance, and repairs.

1. Manufacturing

Sectors 31-33. Activities of this sector are the mechanical, physical, or chemical transformation of materials, substances, or components into new products.

1. Wholesale Trade

Sector 42. Activities of this sector are selling or arranging for the purchase or sale of goods for resale; capital or durable nonconsumer goods; and raw and intermediate materials and supplies used in production; and providing services incidental to the sale of the merchandise.

1. Retail trade

Sectors 44-45. Activities of this sector are retailing merchandise, generally in small quantities, to the general public and providing services incidental to the sale of the merchandise.

1. Transportation and Warehousing, and Utilities (herein termed TWU)
   1. Sectors 48–49. Activities of this sector are providing transportation of passengers and cargo, warehousing and storing goods, scenic and sightseeing transportation, and supporting these activities.
   2. Sector 22. Activities of this sector are generating, transmitting, and distributing electricity, gas, steam, and water and removing sewage through a permanent infrastructure of lines, mains, and pipes.
2. Information

Sector 51. Activities of this sector are distributing information and cultural products, providing the means to transmit or distribute these products as data or communications, and processing data.

1. Finance and Insurance, and Real Estate and rental and leasing [herein termed FIRE]
   1. Sector 52. Activities of this sector involve the creation, liquidation, or change in ownership of financial assets (financial transactions) and/or facilitating financial transactions.
   2. Sector 53. Activities of this sector are renting, leasing, or otherwise allowing the use of tangible or intangible assets (except copyrighted works) and providing related services.
2. Professional, Scientific, and Management, and Administrative and Waste Management Services
   1. Sector 54. Activities of this sector are performing professional, scientific, and technical services for the operations of other organizations.
   2. Sector 55. Activities of this sector are holding securities of companies and enterprises to own controlling interest or influence their management decisions, or administering, overseeing, and managing other establishments of the same company or enterprise and typically undertaking the strategic or organizational planning and decision-making role of the company or enterprise.
   3. Sector 56. Activities of this sector are performing routine support activities for the day-to-day operations of other organizations.
3. Educational Services, Health Care, and Social Assistance
   1. Sector 61. Activities of this sector provide instruction and training in a wide variety of subjects.
   2. Sector 62. Activities of this sector are providing health care and social assistance for individuals.
4. Arts, entertainment, Recreation, and Accommodation and Food Services
   1. Sector 71. Activities of this sector are operating or providing services to meet their patrons varied cultural, entertainment, and recreational interests.
   2. Sector 72. Activities of this sector are providing customers with lodging and/or preparing meals, snacks, and beverages for immediate consumption.
5. Other Services, except Public Administration
   1. Sector 81. This sector provides services not specified elsewhere, including repairs, religious activities, grantmaking, advocacy, laundry, personal care, death care, and other personal services.
6. Public Administration
   1. Sector 92. Activities of this sector are administration, management, and oversight of public programs by Federal, State, and local governments.
